# Metabolic and bariatric surgery reduces aging biomarkers in patients with obesity, independently of achieving optimal total weight loss

**DOI:** 10.20452/wiitm.2025.17946

**Published:** 2025-04-17

**Authors:** Alicja Dudek, Barbara Zapała, Ilona Kawa, Karol Ciszek, Piotr Tylec, Katarzyna Cyranka, Michał Wysocki, Piotr Major

**Affiliations:** Second Department of General Surgery, Jagiellonian University Medical College, Kraków, Poland; Endocrinology Department, Centre of Postgraduate Medical Education, Bielański Hospital, Warszawa, Poland; Department of Pharmaceutical Microbiology, Jagiellonian University Medical College, Kraków, Poland; University Hospital, Kraków, Poland; Department of Psychiatry, Jagiellonian University Medical College, Kraków, Poland; Department of Metabolic Diseases Jagiellonian University Medical College, Kraków, Poland; Department of General Surgery and Surgical Oncology, Ludwik Rydygier Memorial Hospital in Krakow, Kraków, Poland

**Keywords:** aging, bariatric clinical effects, biological age markers, obesity

## Abstract

**INTRODUCTION:**

Obesity, a chronic disease linked to premature aging, is increasingly managed through metabolic and bariatric surgery (MBS).

**AIM:**

This study aimed to evaluate whether changes in biological age markers depended on the optimal weight loss rates resulting from MBS.

**MATERIALS AND METHODS:**

In this prospective, observational study, 100 patients with obesity scheduled for MBS from July 2020 to May 2021 underwent a 24-month postoperative follow-up. The telomere length (TL) was assessed using quantitative polymerase chain reaction. We also evaluated DNA damage, C-reactive protein (CRP), interleukin-6 (IL-6), and tumor necrosis factor α (TNF-α) levels, total oxidant status (TOS), and the metabolic age. We checked whether the percentage of total body weight loss (%TWL) and the percentage excess weight loss (%EWL) correlated with the changes in aging markers postsurgery.

**RESULTS:**

Forty patients completed follow-up, with 22 achieving optimal (%TWL ≥20%; %EWL ≥50%) and 18 suboptimal (%TWL <⁠20%; %EWL <⁠50%) surgical outcomes. Both groups showed a significant TL increase and a reduction of DNA damage, CRP, IL-6, and TNF-α levels, TOS, and the metabolic age. A greater change, however, was only observed for the metabolic age in the optimal outcome group. The Δ of change in the other aging markers did not differ between the groups.

**CONCLUSIONS:**

Improvements in aging markers, such as TL, DNA damage, inflammatory parameters, and TOS occur independently of weight loss rates after MBS, suggesting that weight loss indices alone do not fully capture the therapeutic success of the procedure.

## INTRODUCTION

The rising prevalence of obesity is generating significant public concern due to its association with an increased incidence of severe cardiovascular events, diabetes, cancers, and other systemic diseases.^1^ Obesity is a chronic, recurrent disease characterized by a state of low-grade inflammation that causes premature aging.^2^**^,^**^3^ It has been shown that patients with obesity have shorter telomeres, genome instability, increased inflammation, and elevated oxidative stress, all of them being known markers of aging.^4^**^,^**^5^

Metabolic and bariatric surgery (MBS) is currently the most effective intervention for achieving substantial and sustained weight loss, as well as significantly improving or even inducing remission of associated comorbidities. Studies^6^^,^^7^^,^^8^ demonstrated that in patients who underwent bariatric surgery, a 29% reduction in all-cause mortality was observed, as compared with nonsurgical patients, over a long follow-up period. Emerging evidence^9^ indicates that MBS not only significantly reduces all-cause mortality, but also potentially enhances overall survival and longevity. This effect may be mediated through improvements in biomarkers of biological aging and alterations in molecular pathways implicated in the aging process.^10^**^,^**^11^

Numerous studies^12^**^,^**^13^ have documented the therapeutic success of MBS, typically using numerical weight loss metrics to quantify outcomes. However, those metrics often fail to capture changes in comorbidities, improvements in other objective health parameters, or reductions in mortality rates. A growing body of evidence^14^**^,^**^15^ emphasizes the need to use multivariable mixed models as more accurate methods of assessing clinical effect, instead of the percentage of excess weight loss (%EWL) or the percentage of excess body mass index loss. In addition, it is unclear whether the improvement in aging parameters under MBS is directly related to weight loss, or whether there are other mechanisms that promote antiaging effects of the surgery. Other works^16^**^,^**^17^ also support the hypothesis that metabolic surgery has other weight-independent beneficial effects.

## AIM

The aim of our study was to evaluate whether the change in biological age markers depended on bariatric surgery success, as assessed by weight loss rates.

## MATERIALS AND METHODS

In this prospective, observational study, a cohort of 100 patients with obesity scheduled for MBS at an academic bariatric surgery center between July 2020 and June 2021 was enrolled. A 24-month postoperative follow-up was established. All participants engaged in an extensive postoperative care program which encompassed dietary and psychological support, including a standard balanced diet.^18^**^,^**^19^

Inclusion criteria comprised body mass index (BMI) above 40 kg/m^2^ or above 35 kg/m^2^ with obesity-related complications, and age ranging from 18 to 60 years. Exclusion criteria were reversal bariatric surgery or previous bariatric surgery, pregnancy during the follow-up period, uncontrolled thyroid disorders, kidney diseases, pancreatitis, cancer, depression, neurological disorders, acute inflammatory symptoms or autoimmune disorders based on medical interview and medical history, any condition affecting cognitive function, or taking medications impairing cognitive function.

Medical hisoty, anthropometric parameters, and body composition were assessed in all patients. Biological age markers, including telomere length (TL), DNA damage, levels of interleukin-6 (IL-6), C-reactive protein (CRP), tumor necrosis factor α (TNF-α), total oxidant status (TOS), and the metabolic age were evaluated before and 24 months after surgery.

The patients who completed follow-up and met the inclusion criteria were assessed for the clinical effects of MBS, using standard metrics of clinical evaluation, such as the percentage of total weight loss (%TWL) calculated as (baseline weight – follow-up weight)/baseline weight] and %EWL determined as [(preoperative weight – weight on follow-up)/(preoperative weight − ideal body weight)] × 100. Weight at BMI of 25 kg/m² was considered ideal.

Patients were divided into 2 groups based on whether they achieved optimal (%TWL ≥20%; %EWL ≥50%) or suboptimal (%TWL <⁠20%; %EWL <⁠50%) outcomes.^20^ Both groups were examined for a significant reduction of aging hallmarks following MBS. Finally, a statistical analysis was performed to determine whether the Δ of change in biological age markers differed between the groups.

### Biological age markers

#### Telomere length

Genomic DNA was extracted from blood samples using the NucleoSpin Blood Purification Kit (Macherey-Nagel, Düren, Germany) according to the manufacturer’s protocol. The extracted DNA was subsequently dissolved in an EB buffer. DNA concentration was determined using a Qubit fluorometer (Thermo Fisher Scientific, Waltham, Massachusetts, United States), and DNA integrity was evaluated using an Agilent 2100 Bioanalyzer (Agilent Technologies, Inc., Salta Clara, California, United States).

Absolute TL values were determined using digital polymerase chain reaction (PCR) technology. High-molecular-weight DNA was diluted with molecular-grade water to a concentration of 1 ng/2 µl, and 1 ng of DNA was used as the input for analysis. All samples were measured in triplicate on the same plate and then averaged. PCR amplifications were performed using the Qiagen Qiacuity Digital PCR Machine (Qiagen, Manchester, United Kingdom), following a thermal profile adapted from the quantitative PCR method developed by Cawthon,^18^ with specific modifications (details available upon request).^21^ Each plate included a nontemplate control, along with 3 established reference DNA controls run in triplicate as positive controls.

The forward and reverse telomere primer sequences were adapted from Cawthon^18^ and were as follows: forward: 5′-cggtttgtttgggttt-gggtttgggtttgggtttgggtt-3′ and reverse: 5′-ggcttgccttacccttacccttacccttacccttaccct-3′.

#### DNA damage

Peripheral blood samples were collected from all study participants into ethylenediaminetetraacetic acid tubes and subsequently frozen at −80 °C until the 8-hydroxy-2-deoxyguanosine (8-OHdG) analysis was performed. DNA was extracted using asy Blood and Tissue Kit (Qiagen) and quantified with a NanoDrop 1000 Spectrophotometer (Thermo Fisher Scientific). The 8-OHdG levels were assessed using the EpiQuick 8-OHdG DNA Damage Quantification Direct Kit (Epigentek, Cambridge Bioscience Ltd., Cambridge, United Kingdom), adhering to the manufacturer’s instructions. Absorbance at 450 nm was measured, and values for each sample were calculated based on calibration sigmoid plots with 8-OHdG standards at varying concentrations. The results were presented as a relative quantification against a positive control provided with the kit, and normalized to the input DNA (pg) using the specified formula.

**TABLE 1:** Changes in weight loss rates after surgery in the patients who completed the follow‑up

Variable		All patients (n = 40)
ΔWeight, kg	Median (IQR)	–28.1 (–44.08 to –17.35)
	Range	–75.5 to –8.7
ΔBMI, kg/m²	Mean (SD)	–11.01 (5.7)
%TWL	Mean (SD)	24.08 (12.04)
	Range	54.2–5.8
%EWL	Median (IQR)	52.65 (78.99–30.67)
	Range	164.14–11.28

Abbreviations: BMI, body mass index; %EWL, percentage of excess weight loss; IQR, interquartile range; %TWL, percentage of total weight loss

**TABLE 2:** Characteristics of the study groups

Variable		Group 1, %TWL <20% (n = 18)	Group 2, %TWL ≥20% (n = 22)	*P* value
Age before surgery, y, median (IQR)		42.5 (39.2–52.5)	43 (35.25–49)	0.67
Sex, n (%)	Men	6 (33.3)	8 (36.4)	>0.99
	Women	12 (66.67)	14 (63.6)	
Initial weight, kg, median (IQR)		126.35 (121.75–140.75)	121.5 (110.5–146.5)	0.29
Initial BMI, kg/m², mean (SD)		46.35 (5.01)	44.13 (6.25)	0.46
Body fat mass, kg, median (IQR)		62.25 (53.76–73.08)	56.3 (50.15–67.22)	0.51
Metabolic syndrome, n (%)	Yes	9 (50)	9 (40.9)	0.8
	No	9 (50)	13 (59.1)	
Current smoker, n (%)	Yes	3 (16.67)	6 (27.27)	0.68
	No	15 (83.3)	16 (72.73)	
Type of procedure, n (%)	LSG	17 (94.44)	21 (95.45)	>0.99
	LRYGB	1 (5.56)	1 (4.55)	

A P value <0.05 was considered significant.

Abbreviations: LRYGB, laparoscopic Roux‑en‑Y gastric bypass; LSG, laparoscopic sleeve gastrectomy; others, see TABLE 1

#### Inflammatory paramete rs: interleukin 6, C-reactive protein, and tumor necrosis factor α

In all participants, blood samples were drawn from the cephalic vein following an overnight fast. The samples were centrifuged at 3000 revolutions per minute for 15 minutes to separate the serum, which was then stored at –80 °C. Prior to the analysis, the serum samples were further centrifuged at 10 000 × g for 10 minutes at 4 °C to eliminate any insoluble material.

The concentrations of IL-6 and CRP in serum were quantified using commercially available enzyme-linked immunosorbent assay (ELISA) kits (Bio-Techne, McKinley, Minneapolis, United States). All the procedures were conducted in accordance with the assay protocols and manufacturer’s guidelines. The IL-6 and CRP levels were reported in pg/ml and mg/l, respectively. The serum levels of TNF-α were determined using the Human TNF-α ELISA Kit (Elabscience Bionovation Inc., Houston, Texas, United States), following the manufacturer’s instructions. After completing all procedural steps, the mean (SD) absorbance of triplicate samples was calculated. Data analysis was performed using GraphPad Prism version 7.0 (GraphPad Software Inc., La Jolla, California, Unites States) to determine TNF-α serum concentrations. The results were obtained by reading the plate at 450 nm, and calculated based on the standard curve.

#### Total oxidant status

The procedure followed the instructions included in the TOS Colorimetric Assay Kit (Elabscience). Each sample was analyzed in triplicate. The serum TOS was assessed by measuring the conversion of ferrous ions to ferric ions in the presence of various oxidative substances under acidic conditions. Xylenol orange served as an indicator to detect the rise in ferric ions, indicating the TOS. Calibration of the assay was performed using a hydrogen peroxide (H_2_O_2_) standard. TOS results were reported in μmol/l of H_2_O_2_ equivalent.

#### Metabolic age

Metabolic age was calculated by comparing an individual’s basal metabolism to the average metabolic rate expected for someone of the same age, using the Tanita BC-601 Body Composition Analyzer (Tanita Corp., Tokio, Japan) which incorporates a specific algorithm for this purpose.^22^

### Statistical analysis

Continuous variables were summarized using the number of patients, mean with SD, or median with interquartile range. In the case of categorical data, the frequency distribution of individual responses was presented using the counts of each category and their distribution expressed as percentages. Data were tested for normal distribution with the Shapiro–Wilk test, and for the homogeneity of variances using the Bartlett test. In order to compare selected variables by time point, depending on the assumptions, the paired *t* tests (parametric tests) or Wilcoxon tests were performed. A *P* value below 0.05 was considered significant, but significant results were also indicated for *P* = 0.01 and *P* = 0.001.

**FIGURE 1:**
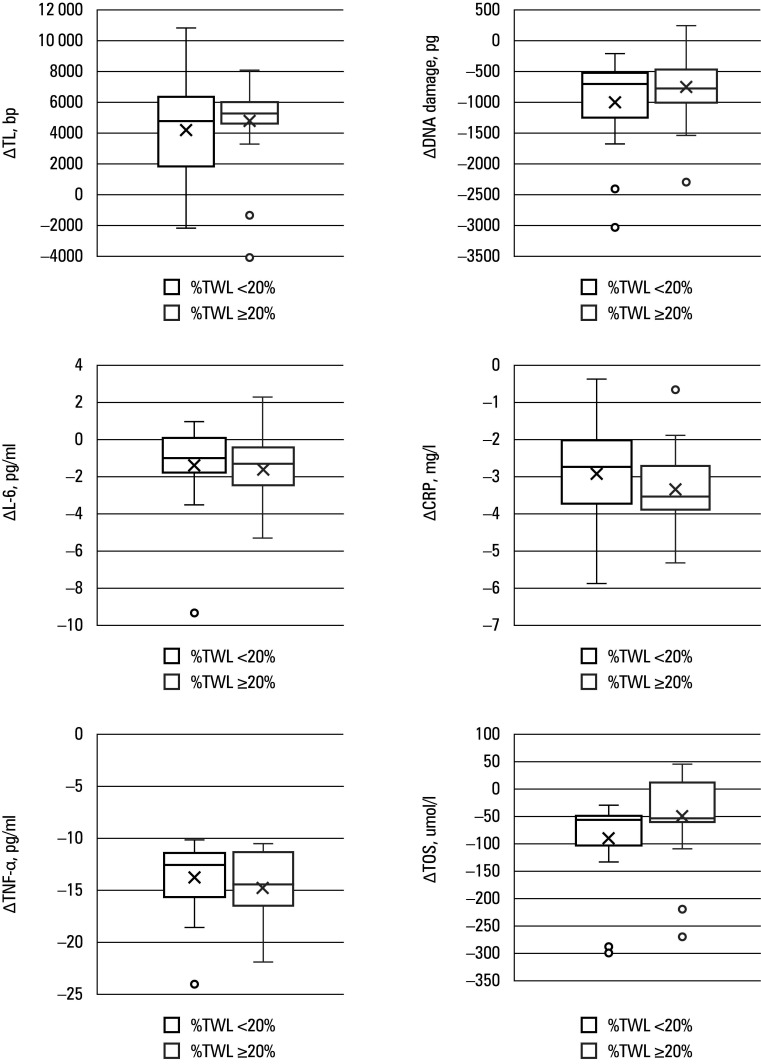
Box plot showing the Δ of change in biological age markers for patients with %TWL below 20% and %TWL equal to or greater than 20%. The boxes represent the IQR, from the 25th to 75th percentiles of the data. The horizontal line inside each box indicates the median value. The crosses represent the mean value. Whiskers extend to the minimum (1st quartile – 1.5 × IQR) and maximum (3rd quartile + 1.5 × IQR) values. Circles denote individual outliers that fall outside this range.

Abbreviations: CRP, C‑reactive protein; IL‑6, interleukin‑6; TL, telomere length; TOS, total oxidant status; TNF-α, tumor necrosis factor α; others: see TABLE 1

### Ethics

All the procedures presented in the study involving human participants were performed under the ethical standards of the institutional and national research committee and in accordance with the 1964 Helsinki Declaration and its amendments, or comparable ethical standards. The study was approved by the Bioethics Committee of the Jagiellonian University (1072.6120.201.2019). Written informed consent was obtained from all the study participants.

## RESULTS

A total of 40 patients met the inclusion criteria and completed the follow-up. Loss to follow-up occurred due to various reasons, including nonattendance at scheduled appointments, pregnancy during the postoperative period, and the need for reoperation or other surgical interventions within the 24-month follow-up period.

TABLE 1 shows the achieved weight reduction effects following MBS for all patients during follow-up.

Out of the 40 patients, 18 (group 1) were described as suboptimal clinical responders, having achieved less than 20% of %TWL and 50% of %EWL. The remaining 22 participants (group 2) were classified as having achieved an optimal clinical effect, reaching a %TWL of 20% or more and a %EWL of 50% or more.

Demographic characteristics of the study groups are summarized in TABLE 2. The average initial BMI values were similar in both groups and the initial body weights were comparable. The sex distribution was also similar in both groups.

**TABLE 3:** Changes in biological age markers before and 24 months after metabolic and bariatric surgery in the study groups

Variable	Group 1, %TWL <20% (n = 18)			Group 2, %TWL ≥20% (n = 22)		
	Before surgery	24 months after surgery	*P* value	Before surgery	24 months after surgery	*P* value
TL, bp, median (IQR)	4084 (3702.25–4285.75)	9300 (7146–9969)	<0.001	4069 (3209–4443.75)	9384 (8853–10773)	<0.001
DNA damage, pg, median (IQR)	1376.9 (1126.64–1636.22)	611.2 (522.28–707.26)	<0.001	1518.15 (1198.23–1983.64)	804.61 (634.68–1028.67)	<0.001
IL-6, pg/ml, median (IQR)	3.49 (2.45–4.52)	2.94 (2.2–3.71)	0.03	4.45 (3.14–5.74)	3.18 (2.42–4.08)	0.007
CRP, mg/dl, median (IQR)	5.64 (5.2–6.15)	3 (2.47–3.56)	<0.001	6.09 (5.59–6.49)	2.83 (2.3–3.28)	<0.001
TNF-α, pg/ml, median (IQR)	23.17 (21.39–25.18)	10.26 (9.75–10.8)	0.03	24.96 (22.97–26.55)	10.79 (9.75–11.72)	<0.001
TOS, μmol/l, median (IQR)	246.5 (189–280.5)	179.5 (145–209.5)	<0.001	199 (193.75–249.75)	203 (144.75–221)	<0.001
Metabolic age, y, mean (SD)	85.25 (16.84)	73.59 (18.02)	<0.001	85.55 (17.04)	62 (17.77)	<0.001

A P value <0.05 was considered significant.

Abbreviations: see TABLE 1 and FIGURE 1

**TABLE 4:** Intergroup comparisons of Δ of change in biological age markers

Variable		All patients (n = 40)	Group 1 <20% (n = 18)	Group 2 ≥ 20% (n = 22)	Test	*P* value
ΔTL, bp	n	40	18	22	Mann– Whitney	0.51
	Median (IQR)	5094.5 (4049.75–6053.75)	4901.5 (2816.25–6308)	5310.5 (4784.75–5942.5)		
ΔDNA damage, pg	n	36	16	20	Mann– Whitney	0.84
	Median (IQR)	–710.4 (–1032.9 to –539.18)	–683.65 (–1055 to –539.18)	–759.07 (–1011.49 to –467.46)		
ΔIL-6, pg/ml	n	36	16	20	Mann– Whitney	0.29
	Median (IQR)	–1.03 (–1.68 to 0.01)	–0.74 (–1.13 to 0.07)	–1.1 (–1.99 to –0.39)		
ΔCRP, mg/l	n	36	16	20	t test	0.1
	Mean (SD)	–2.93 (0.99)	–2.62 (1.02)	–3.17 (0.92)		
ΔTNFα, pg/ml	n	36	16	20	t test	0.23
	Mean (SD)	–13.75 (2.53)	–13.19 (2.12)	–14.2 (2.79)		
ΔTOS, μmol/l	n	36	16	20	Mann– Whitney	0.19
	Median (IQR)	–54 (–54 to –33.25)	–54 (–61.75 to –49)	–54 (–54 to 13.5)		
Metabolic age, y	n	40	18	22	t test	0.01
	Mean (SD)	–18.19 (15.91)	–11.65 (11.09)	–23.55 (17.44)		

A P value <0.05 was considered significant.

Abbreviations: see FIGURE 1 and TABLE 1

First, the changes in biological age markers resulting from the surgery were checked in group 1 and group 2. In both groups, the surgical intervention significantly reduced all the measured markers of aging. TABLE 3 shows the change in biological age parameters following the surgery.

Subsequently, we compared whether the achieved **Δ** of change in the biological age markers following MBS differed between the groups. The results are visualized in FIGURE 1.

The only significant difference in the **Δ** of change in the aging markers was recorded for the metabolic age (TABLE 4). As expected, patients with %TWL greater than or equal to 20% achieved a greater reduction in the metabolic age than those with %TWL below 20% (–23.55 years vs –11.65 years; *P* = 0.01). None of the other parameters (TL, DNA damage, CRP, IL-6, and TNF-α levels, and TOS) differed between the groups. TABLE 5 outlines detailed changes in the aging markers of the groups.

## DISCUSSION

The role of MBS in weight reduction, improving metabolic parameters, and reducing comorbidities is well established.^23^**^,^** ^24^ However, the definition of optimal surgical outcomes remains debatable. Recently, there has been a shift from focusing solely on weight reduction to considering weight-independent effects.^25^**^,^**^26^ This study aimed to investigate whether reductions in the aging markers following MBS differed based on established definitions of bariatric success.

**TABLE 5:** Detailed changes in the aging markers of the groups before and after metabolic and bariatric surgery

Variable	Group 1, %TWL <20% (n = 18)						Group 2, %TWL ≥20% (n = 22)				
		Before surgery (n = 22)	24 months after surgery (n = 22)	Difference	Test	*P* value	Before surgery (n = 18)	24 months after surgery (n = 18)	Difference	Test	*P* value
TL, bp	n	22	22	22	Wilcoxon	<0.001	18	18	18	Wilcoxon	<0.001
	Mean (SD)	4055.36 (1217.65)	8871.82 (2755.88)	4816.45 (2743.21)			3984.28 (718.84)	8187.33 (3743.67)	4203.06 (3750.86)		
	Median (IQR)	4069 (3209–4443.75)	9384 (8853–10773)	5310.5 (4784.75–5942.5)			4084 (3702.25–4285.75)	9300 (7146–9969)	4901.5 (2816.25–6308)		
DNA damage, pg	n	20	20	20	Wilcoxon	<0.001	16	16	16	Wilcoxon	<0.001
	Mean (SD)	1569.83 (560.33)	833.7 (240.59)	–756.13 (598.73)			1471.62 (524.9)	627.77 (225.59)	–843.85 (521.83)		
	Median (IQR)	1518.15 (1198.23–1983.64)	804.61 (634.68–1028.67)	–759.07 (–1011.49 to –67.46)			1376.9 (1126.64–1636.22)	611.2 (522.28–707.26)	–683.65 (–1055 to –539.18)		
IL-6, pg/ml	n	20	20	20	Wilcoxon	0.007	16	16	16	Wilcoxon	0.03
	Mean (SD)	4.67 (2.02)	3.37 (1.08)	–1.29 (1.87)			4.06 (2.4)	3 (1.02)	–1.06 (2.41)		
	Median (IQR)	4.45 (3.14–5.74)	3.18 (2.42–4.08)	–1.1 (–1.99 to –0.39)			3.49 (2.45–4.52)	2.94 (2.2–3.71)	–0.74 (–1.13 to 0.07)		
CRP, mg/l	n	20	20	20	Wilcoxon	<0.001	16	16	16	Wilcoxon	<0.001
	Mean (SD)	6.07 (0.64)	2.89 (0.72)	–3.17 (0.92)			5.74 (0.63)	3.12 (0.94)	–2.62 (1.02)		
	Median (IQR)	6.09 (5.59–6.49)	2.83 (2.3–3.28)	–3.44 (–3.76 to –2.71)			5.64 (5.2–6.15)	3 (2.47–3.56)	–2.65 (–3.35 to –2.01)		
TNFα, pg/ml	n	20	20	20	Wilcoxon	<0.001	16	16	16	Wilcoxon	<0.001
	Mean (SD)	24.86 (2.57)	10.67 (1.55)	–14.2 (2.79)			23.54 (2.5)	10.35 (0.92)	–13.19 (2.12)		
	Median (IQR)	24.96 (22.97–26.55)	10.79 (9.75–11.72)	–14.55 (–15.41 to –11.65)			23.17 (21.39–25.18)	10.26 (9.75–10.8)	–12.77 (–14.17 to –11.81)		
TOS, μmol/l	n	20	20	20	Wilcoxon	0.006	16	16	16	Wilcoxon	<0.001
	Mean (SD)	218.25 (38.75)	187.8 (43.73)	–30.45 (46.75)			241.69 (44.13)	179.25 (42.57)	–62.44 (29.32)		
	Median (IQR)	199 (193.75–249.75)	203 (144.75–221)	–54 (–54 to 13.5)			246.5 (189–280.5)	179.5 (145–209.5)	–54 (–61.75 to –49)		
Metabolic age, y	n	22	22	22	t test	<0.001	18	18	18	t test	<0.001
	Mean (SD)	85.55 (17.04)	62 (17.77)	–23.55 (17.44)			85.25 (16.84)	73.59 (18.02)	–11.65 (11.09)		
	Median (IQR)	83.5 (73.5–94.5)	59.5 (48.25–68.25)	–20.5 (–33 to –9.75)			83 (75.5–95.35)	73.5 (61.25–86.17)	–12 (–15.58 to –4.5)		

A P value <0.05 was considered significant.

Abbreviations: see TABLE 1 and FIGURE 1

We demonstrated that patients achieving both %TWL greater than or equal to 20% and %TWL below 20% showed significant reductions in the aging markers, with no difference in the **Δ** of change between the groups. This indicates that the reduction in the aging markers following MBS occurs independently of weight loss. Additionally, we assessed various aging markers, including TL, DNA damage, inflammatory parameters, and TOS. Only the metabolic age marker correlated with %TWL, as expected due to its direct dependence on body weight.

There are a few studies^27^^,^^28^^,^^29^ evaluating the effect of weight loss on the reduction of the aging markers following surgery, but they describe weight-loss–independent effects on other mechanisms. The almost immediate effect of surgery on the mechanism of glycemic control in patients with type 2 diabetes points to gut hormones and neuroendocrine changes that occur rapidly after surgery, regardless of BMI.

**FIGURE 2:**
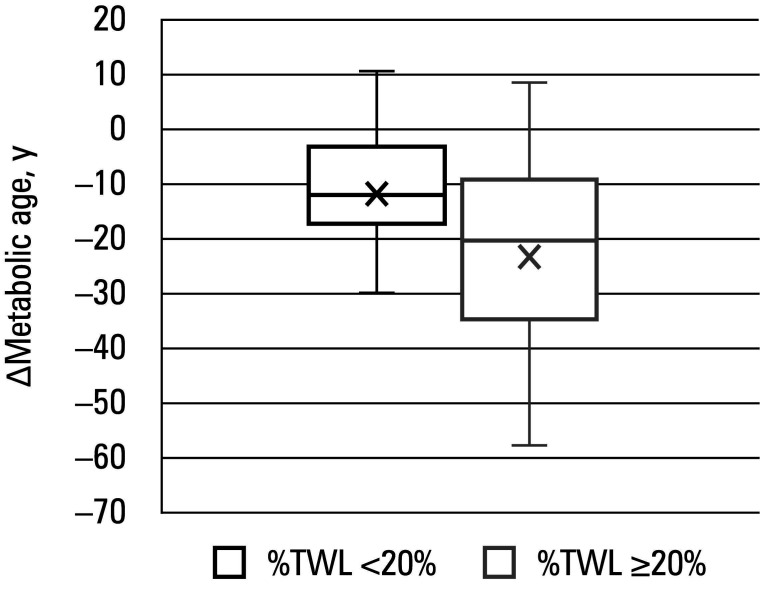
Box plot showing the Δ of change in the metabolic age for patients with %TWL below 20% and %TWL equal to or greater than 20%. The boxes represent the IQR, from the 25th to 75th percentiles of the data. The horizontal line inside each box indicates the median value. The crosses represent the mean value. Whiskers extend to the minimum (1st quartile – 1.5 × IQR) and maximum (3rd quartile + 1.5 × IQR) values.

Abbreviations: see TABLE 1

The effect of MBS on TL remains controversial, but long-term TL increase has been noted. Our study showed significant TL extension 2 years post-MBS, regardless of whether %EWL of 50% was achieved. In contrast, Rolles et al^10^ found TL elongation in lymphocytes only in patients with a BMI loss greater than or equal to 10 kg/m² over a shorter mean (SD) follow-up of 5.5 (3.9) months, which possibly explains the differing results. Consistent with our findings, a 10-year follow-up study on bariatric surgery patients, performed by Laimer et al^30^ on patients demonstrated a TL increase without a correlation with BMI or %EWL. The authors underscore the significance of baseline cholesterol and triglyceride levels on telomere length. Another study^31^ indicated that leptin level changes significantly impact TL.

Weight loss has been shown to reduce genomic damage; however, this effect is not linearly related to weight loss, but may also depend on the reduction of oxidative stress or inflammation, and may be related to the maintenance of a longer plateau period after weight reduction.^32^ A study by Ferk et al^33^ on alterations in gene stability after surgery showed changes in the DNA repair system already in the first month after the procedure, with only a slight increase in subsequent months. This change dynamics indicates that it is not weight loss alone that affects the repair system.

It is hypothesized that reduced inflammatory marker levels resulting from weight loss lead to TL extension and improved gene stability.^30^ Our results show similar reductions in CRP, IL-6, and TNF-α levels in the patients with both optimal and suboptimal surgical outcomes. BMI alone does not reflect changes in body composition, such as visceral and subcutaneous fat or muscle tissue. Visceral adipose tissue generates pro-inflammatory cytokines, while subcutaneous adipose tissue transplant has been shown in animal models to have a positive effect on glucose control and insulin sensitivity.^11^ Furthermore, maintaining muscle mass may enhance the production of myokines, counteracting adipokines and reducing insulin resistance.^34^**^,^**^35^ Despite highlighting the differences in the %TWL obtained, changes in the patients’ body composition may have been more important.

There is only a handful of studies evaluating changes in pro-oxidant / antioxidant homeostasis. Although a study by Chromańska et al^36^ demonstrated a significant reduction in plasma TOS following surgery, the first significant change occurred as early as 1 month postoperatively, and the effect remained at a similar level in the following months, irrespective of further weight loss. These findings align with our observations that TOS reduction also occurs independently of weight loss.

Although numerous studies^24^**^,^**^30^ have shown that surgery can reduce aging markers, the underlying mechanisms are still not fully understood. Weight loss alone is not an accurate parameter to assess the effect of surgery. Aminian et al^37^ showed that a reduction in cardiovascular events occurred in nonsurgical patients at a 20% weight loss, while in surgical patients, at a 10% weight loss. Research focusing on changes in body composition, such as fat distribution and muscle mass, could shed some light on how the reduction of aging hallmarks occurs. In the evaluation of surgical effects, it is worth considering the reduction of aging markers as a positive treatment outcome.

Our study has certain notable limitations. Although we carefully selected a follow-up group receiving aftercare from a dietitian and psychologist, we cannot rule out the impact of medication and supplements on the aging markers measured. Additionally, the small size of the compared groups and follow-up challenges affected our analysis. However, we did make efforts to determine precise exclusion criteria and conduct long-term follow-up. Including an assessment of postoperative physical activity would have further substantiated the study.

## CONCLUSIONS

MBS leads to improvements in aging-related markers, including TL, a reduction in DNA damage, lower levels of inflammatory parameters, and a decrease in TOS. Notably, these benefits occur independently of achieving optimal weight loss.

## JOURNAL INFORMATION

Videosurgery and Other Miniinvasive Techniques is an official journal of the Videosurgery Foundation.
